# Public Hospitals and Their Maintenance

**Published:** 1911-03-25

**Authors:** R. Chambers Norman

**Affiliations:** Secretary-Superintendent of the Alfred Hospital, Melbourne.


### SPECIAL INSTITUTIONAL ARTICLES.

### I.—PUBLIC HOSPITALS AND THEIR MAINTENANCE.

By R. CHAMBERS NORMAN, Secretary-Superintendent of the Alfred Hospital, Melbourne.

(Lecture delivered at the Independent Brotherhood Hall, Melbourne, on Tuesday, August 16, 1910.)

My apology for venturing to address you on "Public hospitals and their maintenance" is that I have been intimately associated with these institutions for thirty years, as in the year 1880, when a "new chum" in Australia, I was appointed Secretary of the Adelaide Children's Hospital, and from that time onwards I have been in close touch with more than one of our Australian hospitals. Until public interest is thoroughly aroused in the management and maintenance of our hospitals, the present unsatisfactory condition of affairs is likely to continue, namely, ad miserecordiam appeals for help first from one institution, then from another; progress hampered; efficiency impaired; while wasteful and obsolete methods of working has to be adhered to, because funds for bringing and keeping things up-to-date are not forthcoming. There is no doubt that hospitals resemble men-of-war in that the type gets out-of-date, but, unlike an obsolete cruiser or battleship, you can't "scrap" a hospital. What was considered a model hospital twenty or thirty years ago is far removed from that now. Take one feature, namely, sanitation! Under the old system it was quite right to put the lavatories as far away as possible, but with our present sewerage system things are different, and now it is nothing short of waste of time and energy that vessels have to be carried to and fro over a distance of sixty yards, as is the case at one hospital not a hundred miles from here. Then, again, what were deemed excellent methods of heating, lighting, and hot water supply formerly are now found to be extravagant and inefficient.

Where space is no object I have a predilection for tents, particularly for the treatment of fever, consumption, or foul surgical cases. The initial cost is not heavy, and the canvas can be cheaply renewed when it becomes weather worn or insanitary.

It will be remembered that at the end of the 'eighties, when typhoid was epidemic in and around Melbourne, we had a large encampment in the Alfred Hospital grounds, and many scores of patients were successfully treated under canvas. I doubt if there is any climate in the world where patients can be treated in tents with greater benefit and less discomfort than in Australia. But, of course, in city hospitals the use of tents, if possible at all, must necessarily be very limited.

As regards bed space, I think medical men will endorse my view that in *permanent* buildings it should not be less than 100 superficial and 1,500 cubic feet, but 150 and 2,000 feet respectively would be better. There it is that temporary structures like tents and chalets have the advantage over bricks and mortar.

### Beds.

As to the number of beds under one management, opinions are bound to differ. Of course, other things being equal, the average cost per bed should be less in a large than in a small hospital, so that economy seems to favour the former. The big institution has, however, its drawbacks. For instance, complete supervision and control are more difficult, while the larger the flock the greater the number of black sheep it is likely to contain. However, there seems no immediate danger in Victoria of our hospitals becoming unmanageably large, at all events not in the near future, for our largest is comparatively small alongside some in England, America, and on the Continent. Thus the London Hospital has over nine hundred beds, that is nearly three times the number available at the Melbourne Hospital.

### Medical Staff.

The transition from beds to medical staff is an easy one. It goes without saying that no other factor counts for so much in the status and usefulness of a hospital as the number and quality of its medical and surgical staff.

The wisdom of having a fixed and not too advanced age for the compulsory retirement from active service on the medical and surgical staffs of our hospitals is undoubted, both in the interests of patients and the rising generation of medicos.

Although no system of election could be devised that would satisfy everyone, I consider the latest innovation in the shape of advisory boards constituted of representatives of our University and Hospital Committees is freer from objection than some previous methods. At all events, the day when a man with a whole alphabet of letters after his name, which not one voter in twenty knew the meaning of, could score over one with simply M.D. or M.S. has gone, never to return—which is a blessing. Ever since the establishment of training schools for nurses in connection with our hospitals there has been a steadily increasing stream of young women possessed by the desire to enter them, with the result that the supply has hitherto exceeded the demand. And the reason is not far to seek. In the first place a sound knowledge of the art of sick nursing never comes amiss, no matter what the future career of the trained might be; while armed with a certificate of competence the nurse who possesses the "mens sana in corpore sano" can face the future fearlessly, because no matter in what part of the cvilised world she may find herself she should always be able to make an honourable living.

I am afraid, however, that in many private houses too much is expected of the nurse; that, in fact, she is regarded as a piece of machinery that can be kept going day and night indefinitely.

My advice to young women who are desirous of becoming nurses is, do not do it unless (1) you feel that it is your vocation, and therefore are prepared to devote all your energies to the work, which you must realise includes much that is trying to nerves and temper and most unpleasant to some of the senses; (2) that you have no constitutional weakness, for unless your health is of the robust order the chances are you will not get through your training without a breakdown. The hours on duty of nurses here are very long. In New Zealand hospitals they have introduced an eight-hours system for nurses, and really I can see no insuperable barrier to its adoption in Victoria.

In existing circumstances one nurse to every three occupied beds is a fair proportion to ensure proper attention to patients, so that if a hospital has an average of 150 occupied beds its nursing staff should number 50, and this is approximately the ratio both at the "Melbourne" and "Alfred" Hospitals. We are told that "comparisons are odious," nevertheless there are times when they are both interesting and instructive. I will therefore give you the following statistics compiled from recent returns:

| | Beds. | Nurses |
|---|---|---|
| Melbourne Hospital ... ... ... ... | 322 ... | 100 |
| Alfred Hospital ... ... ... ... | 164 ... | 53 |
| Auckland Hospital ... ... ... ... | 174 ... | 63 |
| Dunedin Hospital ... ... ... ... | 114 ... | 46 |
| Wellington Hospital ... ... ... ... | 204 ... | 72 |

These figures, you see, bear out what I said as to an eight-hour system involving a larger nursing staff. The office of matron is second to none in importance, for she has the organising of the training school, selection of candidates usually, the control and direction of the nursing work, besides in many cases the supervision of all female servants, and responsibility in regard to stores, food, etc. A woman in this position should have nothing to learn from any of her subordinates from a ward sister down to a scullerymaid. If committees fully realised the moral and commercial value to their hospitals of capable, conscientious, and cultured women as matrons they would not only seek far and wide to obtain such, but would pay them well when they got them. In a paper that I read at the Charities Conference in 1890 on "Hospitals and their Administration," I described the ideal matron in the following words: "Your matron should be amiable and yet firm; even-tempered; just, but not harsh; sympathetic, but not too emotional, possessing tact, energy, and administrative ability, backed up by a fund of the sterling commonsense."

In the discussion that followed, a Mr. Solomon from New Zealand said that the chairman of a hospital of which he was a member of committee was deputed when on a visit to England to secure the services of just such a woman as I had described. He succeeded in finding her, but she never became the matron of his hospital, because *he married her.*

And now for a few words as to management. It may be true that "in the multitude of counsellors there is safety," but it is at the cost of promptitude in the disposal of the agenda. There is one peculiar feature about committees that I have noted, namely, that they will do things in their collective capacity that they would never dream of doing as individuals, and I have not yet got a satisfactory explanation of this psychological phenomenon. Mixed committees of men and women are not usual in the management of general hospitals, though I have worked amicably with both sexes, and I am ready to admit that my own does not possess a monopoly of shrewdness or business ability. *I* really think it would be advantageous if some ladies were affiliated to the boards of all hospitals in an advisory capacity, for they could help a matron in many ways, and see things that should be reported on that no man's eyes would ever detect. In passing, just one word about hospital secretaries. There seems to be an idea in the minds of some that any person who can read, write, and cypher correctly can fill such a position. Though an acquaintance with the three R's is essential, a hospital secretary must be something more than a decent scholar in order to be a success. For that position a man should have a knowledge of human nature, commonsense, and tact; these three, but the greatest of these is tact! And in addition to these qualifications he should lay in a large stock of patience, for he will need it all.

And now let me consider the question of hospital patients. It is just here that I expect my opinions may clash with those of some of my audience. I take it that public hospitals exist for the relief of the sick poor, not for the absolutely destitute only, for in the latter case our hospital accommodation is threefold greater than it need be. Poverty is not a fixed quantity, hence it is that discrimination has to be used in deciding whether or not an applicant for treatment is a suitable case for free treatment. Some men earning 40s. a week could well afford to pay doctors' fees, whilst others with twice that income could not. It is not therefore so much what a man earns as what he is compelled to spend that decides his ability to pay a doctor. That our hospitals are abused goes without saying. I suppose it is the same all the world over, and will continue till the dawn of the millennium.

As contributing to the evil of the abuse of charity, I venture the opinion that the comparatively high fees charged by the medical profession in Victoria for attendance and operations drive numbers of patients to our public hospitals. Let us take for example the case of a working man earning his two guineas a week, with a wife and three young children to support. His house rent will be 10s. or 12s. a week; his wife will want a pound a week for food, and extra for clothing, while he has probably to pay a shilling or two a week to reach his work. He becomes ill and his pay stops! He goes to a doctor who charges him half a guinea for a prescription, then to a chemist who charges 2s. or 3s. for medicine. But that is not all, for if a patient has to be visited and resides more than two miles from a doctor, then the latter can charge at the rate of half a guinea extra for every mile or part of a mile beyond that distance, and when a visit has to be paid to a patient between 9 P.M. and 8 A.M., the doctor can demand a double fee irrespective of distance. Turning to operations, our imaginary "working man" is in a still worse plight, for he would have to pay from two to five guineas for the amputation of a finger or toe, while if he or his wife wanted a tumour extirpated, or a major operation performed on the eye, he might be let in for anything from five to a hundred guineas—and these fees do not include after-attendance, or the payment of an assistant or anæsthetist!

The charges I have quoted are simply to show that for the average working man or low-salaried clerk a public hospital must be taken advantage of whenever accident or illness renders him "hors de combat." When I began my hospital work in Adelaide I found such an abuse of the Children's Out-Patient Department that for the first three months I must have refused some 20 per cent. of the applicants, while at the end of a year the percentage of refusals came down to about 10, and subsequently to less than 5.

As to the question—"are hospital patients grateful"? I would reply that in the majority of cases they are moderately so.

One word about so-called "religious visitors"; not the accredited representatives of recognised religious bodies, but those "free-lances" who take advantage of visiting days to distribute tracts, and who possess zeal, no doubt, but not according to knowledge.

For instance, you will find a tract left on the bed of a Roman Catholic patient in which the Scarlet Woman of Revelation is stated to be identical with the Pope. I have been shown a tract with the startling heading "You are going straight to hell," which had been handed to a patient hovering between life and death. Well, now, a printed statement of that sort, even if true, is not a cheerful intimation, and hardly calculated to buck one up for a struggle with disease. And yet, doubtless, the distributor meant well, and thought he or she was doing good work.

For the information of the public I will enumerate what are always acceptable gifts in kind to hospitals and appreciated. Old linen, providing it is quite clean, but not rags, unless the hospital you patronise happens to run a paper mill. Fresh eggs and good sound fruit, particularly lemons. Illustrated papers and magazines that are not too ancient and have not passed through too many hands are acceptable, as are also good books (I do not mean "goody" ones!) when you have not perforce to skip from page 113 to 161. Fresh cut flowers are a real source of delight to most patients, and brighten up a ward or sickroom wonderfully. What a drear and miserable abode this world of ours would be were it not for flowers! Of course such things as water or air beds and cushions, providing they are in good condition, invalid chairs and lounges, crutches, and such-like appliances, come in handy and often save the cost of purchasing.

*(To be continued.)*

